# Programmable *Klebsiella pneumoniae* Phage Tropism Enabled by Scalable Receptor‐Binding Protein Mining and Modular Assembly

**DOI:** 10.1002/advs.202514511

**Published:** 2026-02-19

**Authors:** Shisong Jing, Yiyao Song, Xianbiao Bi, Yuqin Song, Dawei Wei, Jiangqing Huang, Yuan Zeng, Gang Zhang, Rong Zhang, Chao Wang, Jie Feng

**Affiliations:** ^1^ State Key Laboratory of Microbial Diversity and Innovative Utilization Institute of Microbiology Chinese Academy of Sciences Beijing China; ^2^ College of Life Science University of Chinese Academy of Sciences Beijing China; ^3^ School of Clinical and Basic Medical Sciences Shandong First Medical University & Shandong Academy of Medical Sciences Jinan Shandong China; ^4^ Department of Clinical Laboratory Second Affiliated Hospital of Zhejiang University, School of Medicine Hangzhou China

**Keywords:** antimicrobial resistance, capsular tropism, programmable phages, receptor‐binding proteins (RBP), synthetic biology

## Abstract

Phage therapy is an attractive countermeasure to multidrug‐resistant pathogens, but clinical deployment is limited by the narrow and poorly predictable host range of most phages. Public repositories now house tens of thousands of phage genomes, yet there is no systematic route for turning this sequence space into designer phages with defined receptor specificities. Here we present a scalable, data‐driven framework that converts receptor‐binding protein (RBP) diversity into a modular toolkit for programmable phage engineering. Using *Klebsiella pneumoniae* as a model, we mined 280 non‐redundant *Przondovirus* RBP sequences and resolved them into >50 discrete clusters. Functional screening of the Prz_RBPs from 41 previously uncharacterized *Przondovirus* phages expanded the number of experimentally validated capsular locus (KL) targets from 14 to 32 in *Przondovirus*. Each cluster was primarily associated with a dominant KL type, enabling construction of a genotype‐to‐phenotype map that accurately predicts receptor tropism. The identification of conserved anchor motifs enabled combinatorial pairing of Prz_RBP1 and Prz_RBP2 as plug‐and‐play modules for programmable phage tropism. Supplying exogenous Prz_RBP2 variants that assemble with Prz_RBP1 further and predictably expands host range, providing broad and tunable coverage. This framework transforms raw genomic diversity into customizable antibacterial agents and offers a general blueprint for precision phage therapy.

## Graphical abstract

This work introduces a scalable, data‐driven strategy that turns the vast sequence diversity of phage receptor‐binding proteins into modular tools for engineering customizable phages. By clustering and experimentally validating diverse RBPs, the authors build an accurate map linking sequence to host specificity and create plug‐and‐play modules that enable predictable and tunable expansion of phage tropism.

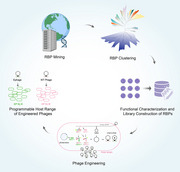

## 1 Introduction

Antimicrobial resistance poses a global health crisis, causing an estimated 4.71 million deaths in 2021, of which 1.14 million were directly due to drug‐resistant infections [1]. Phage therapy offers a highly specific alternative to conventional antibiotics with minimal disruption to the host microbiota [2, 3, 4]. However, its clinical deployment is constrained by the narrow host range of most phages, which typically infect only a few strains within a species [5]. Consequently, candidate phages must be isolated individually and assembled into bespoke libraries, a process that is slow and resource‐intensive [6, 7, 8]. Large databases such as Prophage‐DB, INPHARED, and PhageScope collectively contain millions of phage genomes [9, 10, 11], providing an unprecedented resource for translational applications. Nevertheless, due to the lack of systematic functional annotation and engineering strategies, these resources still cannot be effectively converted into phage candidates with practical application value.

Advances in synthetic biology have provided a feasible approach for systematically mining and exploiting phage genomic resources. Huss et al. built a saturation‐mutagenesis library, mined functional modules and rewired a benign T7 phage into a strain‐targeted therapeutic against *Escherichia coli* O121 [12, 13]. In parallel, we analyzed a collection of phages and identified multiple RBPs, which were subsequently transplanted into other phages to switch or broaden their host range [14]. These studies illustrate that phage genomic data can be systematically analyzed and repurposed for rational design of engineered phages with defined targeting specificity. However, a scalable and coherent framework capable of converting large sequence repositories into readily accessible, target‐specific, programmable phages is still lacking, limiting the translational potential of phage therapy.

Building on advances in synthetic biology and modular receptor‐binding protein (RBP) design, early clinical applications have demonstrated the feasibility of engineered phages. For example, two clinical cases employed engineered phage cocktail therapy, effectively lysing multidrug‐resistant *Mycobacterium* strains in patients with severe cystic fibrosis, achieving sustained clinical improvement without severe adverse events [15, 16]. In addition, Gencay et al. have developed the engineered phage cocktail SNIPR001, which has already entered a phase I clinical trial [17]. These clinical milestones indicate that applying modular design strategies to programmable phages is practically feasible. Nonetheless, rational design of engineered phages still relies on understanding RBP‐mediated molecular interactions, which define host specificity by recognizing bacterial surface receptors [18, 19]. The rapid growth of public sequence databases has resulted in tens of thousands of putative RBP sequences, creating an opportunity to mine these data for modular components. However, programming host specificity via RBP engineering remains a major challenge. Structural compatibility between engineered RBPs and the surrounding tail architecture is difficult to predict, and substitutions frequently lead to misfolded proteins, defective assembly, or loss of binding function [20]. Most existing studies have focused on C‐terminal enzymatic domains, with limited understanding of the N‐terminal anchoring domains, inter‐domain linkers, and terminal tail segments that are essential for modular assembly, conformational regulation, and structural integration [21]. These knowledge gaps continue to obstruct the creation of a truly general, programmable phage platform.

*Klebsiella pneumoniae*, including its carbapenem‐resistant strains (CRKP), is a clinically significant Gram‐negative pathogen responsible for liver abscesses, bacteremia, urinary tract infections, and a range of hospital‐ and community‐acquired infections [22]. The World Health Organization has designated CRKP as a critical priority pathogen due to its increasing resistance to last‐line antibiotics [23]. Effective phage therapy is hindered by extensive capsular heterogeneity: over 160 capsule locus (KL) types encode unique polysaccharides [24, 25], and most phages recognize only one or two types [26]. To date, functional characterization of capsular polysaccharides (CPS)‐targeting RBPs remains limited, only 58 RBPs have been confirmed to exhibit specificity toward 31 KL types [27], and modular incompatibility across phylogenetically distant phages restricts direct substitution [28]. Consequently, *Klebsiella* phages, which are rich in capsular and RBP diversity but constrained by narrow tropism, offer an ideal model system for developing a coherent and scalable strategy to convert vast sequence repositories into accessible, target‐specific, programmable therapeutics.

In this study, we systematically mined publicly available databases for *Klebsiella*‐infecting *Przondovirus* genomes and identified two major RBP classes, Prz_RBP1 and Prz_RBP2. From these, we curated 280 nonredundant representative sequences and organized them into more than 50 distinct sequence clusters, each showing strong correlation with specific *K. pneumoniae* KL types. Based on this, we assembled and validated a modular RBP library that expanded the experimentally verified KL coverage of *Przondovirus* phages from 14 to 32 types. Selected RBP modules were then integrated into a synthetic phage chassis using a conserved T4gp10‐like domain as a universal docking site, enabling plug‐and‐play replacement and multivalent capsule recognition with predictable host switching. In addition, the highly flexible combinatorial architecture between Prz_RBP1 and Prz_RBP2 allows these modules to be supplied from extrachromosomal plasmids with excellent compatibility. These advances establish a scalable, data‐driven framework for converting large sequence repositories into readily accessible, target‐specific, programmable therapeutic phages, addressing a central bottleneck in the field.

## 2 Results

### 2.1 Two Types of RBPs in *Przondovirus* Phage: Prz_RBP1 and Prz_RBP2

**FIGURE 1:**
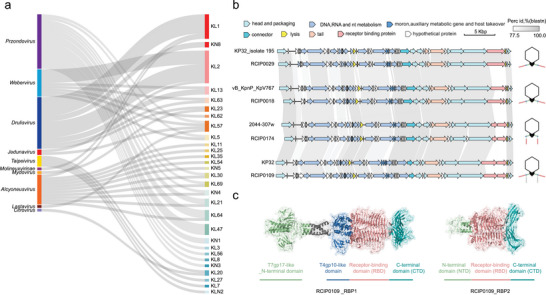
Structural and functional diversity of RBPs in *Przondovirus* phages. (a) Sankey diagram showing the host specificity of experimentally validated RBPs from *K. pneumoniae* phages across different genera, highlighting their distinct recognition of KL capsular types. (b) Whole‐genome alignment of *Przondovirus* phages reveals overall genome conservation, with major divergence localized to RBP‐coding loci. Representative tail fiber architectures are illustrated accordingly. Phages used for alignment include KP32_isolate 195 (MH172263), RCIP0029 (OR532823), vB_KpnP_KpV767 (KX712070), RCIP0018 (OR532812), 2044–307w (MF285615), RCIP0174 (PV854175), KP32 (GQ413937), and RCIP0109 (OR532903). (c) Trimeric structures of RBP1 and RBP2 from phage RCIP0109 predicted by AlphaFold3 reveal domain organization and conserved structural motifs critical for host interaction.

Understanding the taxonomy and distribution of *K. pneumoniae* phages is crucial for developing effective therapeutic strategies. Among these phages, *Przondovirus* stands out as the most abundant genus, with 234 representatives identified in the Genebank Database (1662 genomes as of March 2025, Figure S1a). To date, RBPs from *Przondovirus* have been shown to functionally target 14 distinct capsule types (as confirmed through heterologous expression and activity validation, Table S1), demonstrating a remarkable breadth in host capsule recognition (Figure 1a; Figure S1b). In contrast, the identified RBPs from *Webervirus* and *Drulisvirus* can each cover only six capsule types. This unique combination of prevalence and broad RBP coverage makes *Przondovirus* an ideal candidate for in‐depth exploration of recognition strategies employed by phages to identify and bind to their bacterial hosts.

A comparative genomic analysis of *Przondovirus* phages revealed significant conservation across all genomic regions, except for the genes encoding tail fiber or tail spike proteins (i.e., RBPs), which typically determine host specificity (Figure 1b). We extracted all RBP sequences from these *Przondovirus* genomes and predicted their structures using AlphaFold3. These RBPs can be divided into two types according to their structural characteristics and functional domains, and we designated them as Prz_RBP1 and Prz_RBP2 (Figure 1c). Prz_RBP1 generally comprises four functional domains: (1) a T7gp17‐like N‐terminal domain that connects the protein to the baseplate, (2) a flexible T4gp10‐like domain that serves as an anchor for RBP2, (3) a catalytic domain with a characteristic right‐handed parallel *β*‐helix structure that functions as a depolymerase to recognize and degrade CPS, and (4) a C‐terminal domain that stabilizes trimer formation. By comparison, Prz_RBP2 contains three domains and lacks the conserved T7gp17‐like N‐terminal domain that connects RBP to the baseplate. Notably, Prz_RBP2's N‐terminal anchors to RBP1's T4gp10‐like domain, thereby facilitating its structural association with Prz_RBP1 [29]. Its catalytic domain and C‐terminal domain closely resembles that of RBP1, suggesting a similar mechanism of action in CPS degradation. Some RBPs lacked catalytic domains and were subsequently excluded from further analysis, which were retained, resulting in 280 non‐redundant RBPs for downstream study.

### 2.2 Clustering of RBPs in *Przondovirus* Phages and Its Correlation with Capsule Types

**FIGURE 2:**
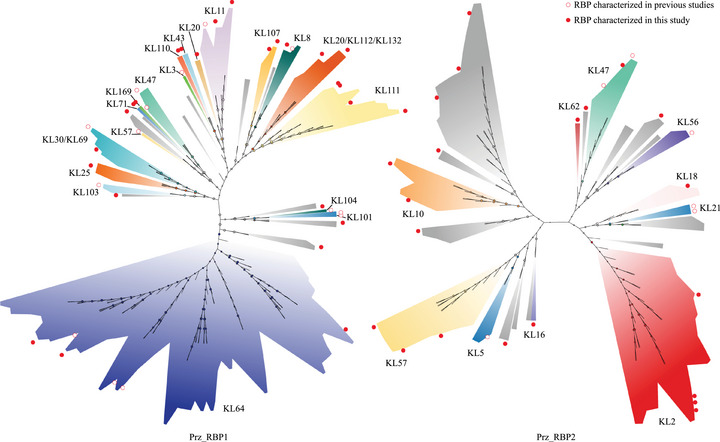
Phylogenetic analysis of Prz_RBP1 and Prz_RBP2 reveals KL type‐specific RBP clades. Maximum likelihood trees were constructed for 280 non‐redundant Prz_RBP sequences. Prz_RBP1 and Prz_RBP2 formed distinct clades, each displaying high intra‐cluster similarity and a strong correspondence to specific KL types. Bootstrap support values (1000 replicates) are shown at the corresponding internal nodes. Branch colors indicate the associated KL types (gray indicates unknown KL types). Clusters with a cool‐colored background contain previously characterized RBPs, while clusters with a warm‐colored background contains RBPs characterized in this study. Previously reported RBPs are marked with red hollow circles, and RBPs selected and characterized in this study are marked with red solid circles.

To further elucidate the phylogenetic classification of Prz_RBPs, we conducted a multiple sequence alignment of 280 non‐redundant Prz_RBP protein sequences using MAFFT, followed by phylogenetic reconstruction. The results indicated that both Prz_RBP1 and Prz_RBP2 formed multiple distinct clusters (Figure 2). High sequence similarity was observed within the clusters, while similarity between adjacent clusters was markedly lower, demonstrating clear evolutionary boundaries among them.

In Prz_RBP1, the 183 RBPs were classified into 29 distinct phylogenetic clusters, ranging in size from 1 to 85 members. Thirteen of these clusters contained three or more members. From the total 29 clusters, 10 contained functionally annotated RBPs (Table S1). Specifically, RBPs from nine clusters targeted nine distinct KL types: KL64, KL103, KL57, KL47, KL3, KL11, KL8, KL101 and KL104. Additionally, one cluster contained an RBP capable of targeting both KL30 and KL69. This clustering pattern indicates a strong correlation between individual RBP clusters in Prz_RBP1 and specific KL types. Notably, the remaining 19 uncharacterized clusters may correspond to novel RBPs associated with previously undefined capsular types. Further characterization of these RBPs could provide critical insights into phage‐host interactions and support the rational design of engineered phages to target diverse KL types.

A similar phylogenetic pattern was observed in Prz_RBP2, where 97 RBPs were assigned to 22 clusters ranging in size from 1 to 22 members. Of these 22 clusters, 11 contained three or more RBPs. Functional annotations were available for four clusters, targeting KL21, KL47, KL56, and KL5 (Table S1). The remaining 18 clusters lacked known functional information. Interestingly, both Prz_RBP1 and Prz_RBP2 encoded RBPs targeting KL47; however, these RBPs exhibit substantial sequence divergence and pronounced structural differences (Figure S2a), suggesting that they may have independently evolved to recognize structurally distinct variants of KL47 capsule polysaccharides [30].

### 2.3 Functional Characterization of RBPs in *Przondovirus* Phages

To investigate the function of uncharacterized RBP clusters, we selected 1 to 4 RBPs from each of 15 unannotated Prz_RBP1 and Prz_RBP2 clusters that contained three or more members, yielding a total of 27 candidate proteins. To further capture the diversity among low‐abundance clusters, we additionally selected one RBP from each of 12 clusters out of 22 that contained fewer than three members for functional characterization. To validate the KL‐RBP associations proposed for previously characterized clusters, we also included 8 RBPs from annotated clusters targeting KL64, KL47, KL30, KL8, and KL11. In total, 47 RBP candidates were selected, of which 41 were successfully expressed in a soluble form, enabling further functional assays and characterization (Figure S3).

**FIGURE 3:**
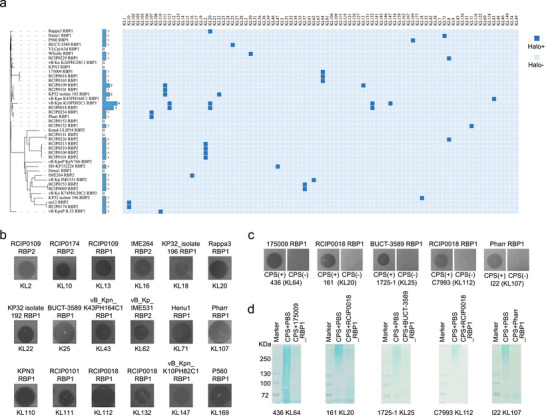
Functional validation of Prz_RBPs and their specificity toward distinct KL capsular types. (a) Interaction matrix showing the activity profiles of 41 solubly expressed Prz_RBPs (columns), ordered according to their phylogenetic relationships, against 174 *K. pneumoniae* clinical isolates (rows), covering 88 distinct KL capsular types. Each square represents a spot assay result; dark blue squares indicate visible halos, and light blue squares indicate absence of halos. A blue bar chart on the left displays the number of KL types showing visible halos for each RBP. (b) Halo formation results showing the KL types targeted by newly characterized Prz_RBPs identified in this study. (c) Spot assay images comparing wild‐type strains and their corresponding CPS‐deficient mutants. Halos were observed on wild‐type strains but not on mutants. (d) SDS‐PAGE analysis of purified CPS incubated with selected Prz_RBPs. CPS degradation was detected after incubation.

To assess host specificity, we established a test panel comprising 174 *K. pneumoniae* clinical isolates representing 88 distinct capsular types (capsule synthesis loci, KL types), including major clinical serotypes such as KL64, KL47, KL107, KL2, KL1, and KL57. It is well established that depolymerases encoded by phage RBPs degrade bacterial CPS, producing clear and stable halos on bacterial lawns—an indicator of depolymerase activity [29, 31]. Using a spot assay, we systematically screened interactions between 41 soluble RBPs and bacterial strains representing 88 KL types (Figure 3a). Among the 41 soluble RBPs tested, 32 exhibited detectable halos on strains representing 24 distinct KL types. These RBPs belonged to 21 distinct RBP clusters, of which 19 were associated with only a single KL type, demonstrating high specificity in capsule recognition. Importantly, among the 19 clusters identified, 5 had been previously reported, while 14 clusters representing novel RBP‐KL type interactions were newly characterized in this study, substantially expanding the known repertoire of functional RBPs. Based on structural features and sequence homology, the novel RBPs were classified into RBP1 family (targeting KL107, KL110, KL111, KL169, KL20, KL25, KL43, KL71, and KL62) and RBP2 family (targeting KL18, KL2, KL16, KL57, and KL10). This systematic characterization substantially expanded the RBP toolbox by identifying 14 novel RBP clusters, while supporting a predictive framework where RBP cluster membership correlates with capsular specificity, enabling rational RBP discovery through bioinformatic approaches.

In addition, we identified two clusters harboring Prz_RBP1s with multi‐capsular targeting capability. RCIP0018_RBP1 and vB_Kpn_K10PH82C1_RBP1 from one cluster exhibited depolymerase activity against KL20, KL112, and KL132, with the latter additionally targeting KL147. Meanwhile, RCIP0101_RBP1, RCIP0109_RBP1, and KP32_isolate 192_RBP1 belong to the same RBP cluster, and all recognize KL111. Notably, RCIP0109_RBP1 and RCIP0111_RBP1 further target KL13 and KL22, respectively. Sequence alignment revealed that these RBPs share over 90% amino acid identity and exhibit nearly identical structural, indicating that subtle sequence variations can lead to differences in host range (Figure S4). Collectively, including these multi‐capsular RBPs, our characterized RBP library provides targeting against 18 additional KL types, substantially expanding the component repertoire for programmable phage engineering (Figure 3b).

To further validate that CPS serves as the direct target of the tested RBPs, we constructed CPS‐deficient mutants for five KL types: KL20, KL25, KL64, KL107, and KL112. Spot assays were then performed on CPS‐present (wild‐type) and CPS‐absent strains to evaluate depolymerase activity. All RBPs produced clear and reproducible halos exclusively on wild‐type strains, while no halo was observed in the CPS‐deficient mutants (Figure 3c). Additionally, CPS was extracted and purified from each strain and subjected to in vitro digestion with the corresponding RBPs. The degradation of CPS was assessed by SDS‐PAGE, and the results showed that CPS was almost completely degraded upon RBP treatment, further confirming that these RBPs utilize CPS as a substrate for their enzymatic activity (Figure 3d).

### 2.4 RBP Cluster‐based Host Prediction Is Broadly Applicable Across Diverse Phages

To assess whether our clustering strategy is applicable to phages from other genera, we extended the analysis to *Drulivirus* RBPs. Among 155 *Drulivirus* phages surveyed, most encoded a single RBP that could be classified as either Dru_RBP1 or Dru_RBP2. Because previous studies have primarily focused on Dru_RBP2, we conducted a phylogenetic analysis of these proteins. We then mapped the functionally characterized Dru_RBPs (Table S1) onto the phylogenetic tree and observed that RBPs targeting different KL types formed distinct clusters. This pattern was consistent with our earlier findings, supporting a one‐to‐one correspondence between RBP clusters and KL types (Figure S5). These results indicate that our RBP mining and clustering framework can be readily generalized to phages from other genera.

To experimentally validate the functional potential of uncharacterized clusters, we synthesized five representative RBP genes from five distinct clusters and evaluated their activities. Spot assays revealed that these RBPs selectively targeted KL51, KL24, KL64, KL39, and KL62, respectively. Together, these findings further demonstrate that our RBP mining and clustering strategy is broadly applicable across diverse phage genera.

### 2.5 Harnessing RBP Modules for Host Range Reprogramming in Chassis Phage RCIP0109

The identified RBP modules provide a comprehensive toolkit for phage engineering, facilitating precise modulation of host range within a unified chassis phage framework. RCIP0109, a representative member of the *Przondovirus* genus, was selected as the chassis phage. Its genome is 41,152 bp in length, with a GC content of 53.05%, and it lacks genes encoding tRNA, antibiotic resistance, toxins, virulence factors, or lysogeny‐promoting gene clusters. RCIP0109 harbors RBP1 and RBP2, both possessing functional depolymerase domains, targeting KL2 and KL111, respectively.

**FIGURE 4:**
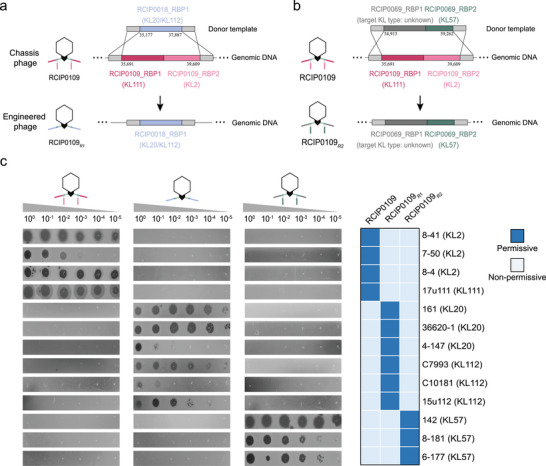
Reprogramming host specificity of chassis phages through modular RBP replacement. (a) Schematic of single RBP module replacement: Both RBP1 (targeting KL111) and RBP2 (targeting KL2) of phage RCIP0109 were replaced with RCIP0018_RBP1, which targets KL20 and KL112, generating the engineered phage RCIP0109*_R1_*. The genomic positions of donor and recipient RBPs are indicated in the diagram. (b) Schematic of dual RBP module replacement: RBP1 and RBP2 from phage RCIP0069 were used to simultaneously replace the native RBPs of RCIP0109, generating the dual‐RBP recombinant phage RCIP0109*_R2_*. (c) Spot assay results validating host range shifts: RCIP0109*_R1_* and RCIP0109*_R2_* lost lytic activity against their original KL2 and KL111 hosts but acquired infectivity toward KL20/KL112 and KL57 strains, respectively.

To reprogram host specificity, we employed homologous recombination to replace both native RBPs of phage RCIP0109 with the RBP1 of phage RCIP0018, which targets KL20 and KL112 (Figure 4a). The resulting engineered phage, RCIP0109*_R1_*, lost the ability to infect KL2 and KL111 hosts but acquired lytic activity against KL20 and KL112 strains, demonstrating successful reprogramming of host specificity (Figure 4c). Next, we constructed the recombinant phage RCIP0109*_R2_* by replacing the two native RBPs of RCIP0109 with the corresponding RBPs from phage RCIP0069, which target an unknown KL type and KL57, respectively. (Figure 4b). The engineered RCIP0109*_R2_* lost activity against KL2 and KL111 but gained the ability to lyse KL57 (Figure 4c). Based on this strategy, we further constructed engineered phages targeting two clinically prevalent *K. pneumoniae* capsular types, KL64 (RCIP0028_RBP1) and KL25 (BUCT‐3589_RBP1), designated as RCIP0109*_R3_* and RCIP0109*_R4_*, respectively. Upon replacement of the RBP modules, these engineered phages lost the ability to infect their original hosts (KL2 and KL111) while acquiring lytic activity against KL64 and KL25, respectively (Figure S6). Collectively, these results demonstrate that RBP modules can be selectively assembled onto chassis phages to achieve modular and predictable reprogramming of host specificity.

### 2.6 Combinatorial Pairing of Prz_RBP1 and Prz_RBP2 Modules Enables Programmable Phage Tropism

Prz_RBP1 contains a T4gp10‐like domain that is proposed to serve as an anchoring platform for Prz_RBP2. In addition, the N‐terminus of Prz_RBP2 harbors a short, conserved amino acid motif that is thought to mediate its interaction with Prz_RBP1 [29, 32]. Based on this architecture, we hypothesized that the T4gp10‐like domain and the N‐terminal motif in Prz_RBP2 together form a modular interface that enables the combinatorial assembly of RBPs from distinct phage sources. To investigate this hypothesis, we first examined the distribution of the T4gp10‐like domain across 183 Prz_RBP1 sequences and found that 84 of them (identity 41.03%–66.67%) contained this domain. These RBPs were distributed across 28 of the 40 identified Prz_RBP1 clusters and collectively covered 19 out of 22 KL types targeted by Prz_RBP1s, suggesting that the T4gp10‐like domain is broadly conserved (Figure S7a). We then analyzed the N‐terminal regions of 97 Prz_RBP2 proteins and identified a ∼30 amino acid conserved motif (identity 37.50%–84.38%, Figure S7b) present in all sequences. Together, these findings support the widespread presence of the two complementary structural elements within the *Przondovirus* RBP repertoire.

**FIGURE 5:**
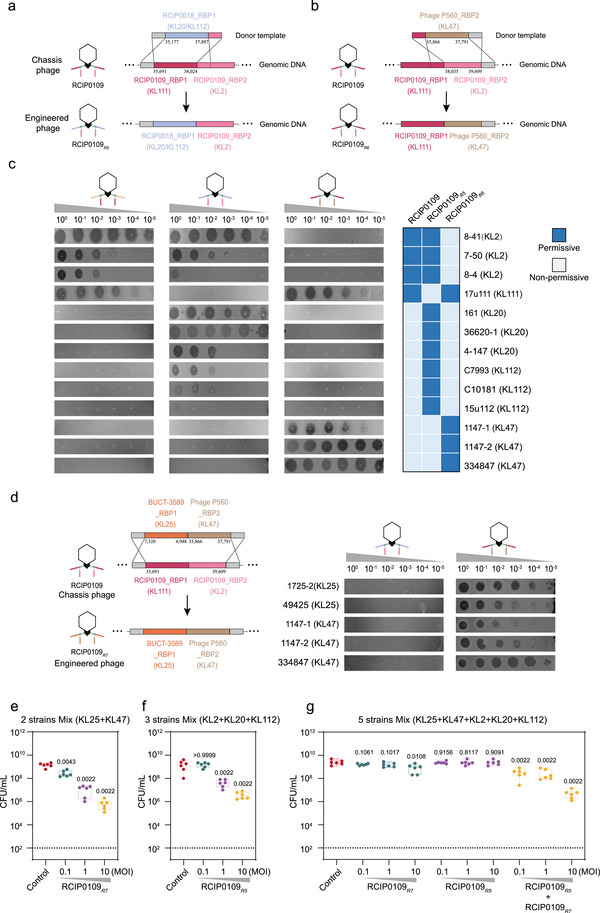
Modular assembly of RBP components mediated by the conserved T4gp10‐like domain in Prz_RBP1. (a) Schematic of RBP1 replacement: RCIP0018_RBP1 (targeting KL20/KL112) was substituted for the native RBP1 (targeting KL111) in RCIP0109, while the original RBP2 (targeting KL2) was retained, yielding the chimeric phage RCIP0109*_R5_*. The genomic positions of donor and recipient RBPs are indicated in the diagram. (b) Schematic of RBP2 replacement: *Kp*‐phage P560_RBP2 (targeting KL47) was introduced in place of the native RBP2, while RBP1 (targeting KL111) remained unchanged, generating RCIP0109*_R6_*. (c) Spot assays confirming the functional compatibility of modular RBP assembly. RCIP0109*_R5_* lysed both KL2 and KL20/KL112 strains, while RCIP0109*_R6_* lysed KL111 and KL47 strains. (d) Schematic representation of RCIP0109*_R7_* construction and host‐range validation. RBP1 from BUCT‐3589 (targeting KL25) and RBP2 from phage P560 (targeting KL47) were combined to replace the native RBPs in the chassis phage, enabling RCIP0109*_R7_* to infect both KL25 and KL47. (e–g) Quantification of CFU at 3 h after treatment with engineered phages at different MOIs. (e) RCIP0109*_R7_* significantly reduced CFU in the KL25 and KL47 mixed cultures. (f) RCIP0109*_R5_* effectively decreased CFU in the KL2, KL20, and KL112 mixed cultures. (g) Individual engineered phages showed limited inhibition against the five‐strain mixed culture, whereas the combination of RCIP0109*_R7_* and RCIP0109*_R5_* resulted in a markedly greater reduction of the mixed bacterial population. Each group was compared to the control, and *p* values are shown above the corresponding box plots. The dashed line indicates the limit of detection.

To functionally validate this modular interaction, we first replaced the native RCIP0109_RBP1 with RCIP0018_RBP1, which targets KL20/KL112, while retaining the native RBP2 (Figure 5a). RCIP0018_RBP1 and RCIP0109_RBP1 shared 71.01% sequence identity in the T4gp10‐like domain and exhibited highly similar tertiary structures (Figure S7c). The resulting recombinant phage, RCIP0109*_R5_*, lost the ability to infect KL111 but gained lytic activity against KL20/KL112 while retaining activity against KL2 (Figure 5c). Next, we replaced the native RCIP0109_RBP2 with P560_RBP2, which targets KL47, while keeping the native RBP1 (Figure 5b). Despite only 53.62% sequence identity between phage P560 RBP1 and RCIP0109_RBP1 in the T4gp10‐like region, the structures were highly similar (Figure S7d). The engineered phage, RCIP0109*_R6_*, lost activity against KL2 but gained lytic activity against KL47 while retaining infectivity for KL111 (Figure 5c). Finally, to test the modular compatibility between RBPs from distinct phages, we co‐assembled the KL25‐targeting BUCT‐3589_RBP1 from phage BUCT‐3589 with the KL47‐targeting P560_RBP2 from phage P560. The resulting phage, RCIP0109*_R7_*, lost its native host range (KL2 and KL111) but simultaneously acquired lytic activity against KL25 and KL47 (Figure 5d). These data demonstrate that Prz_RBP1s harboring the T4gp10‐like domain and Prz_RBP2s containing the conserved N‐terminal motif function as combinable modules, enabling predictable and non‐redundant customization of phage tropism through their free pairing.

As an application‐level validation, we formulated phage cocktails based on engineered phages to demonstrate their precise capsular‐type targeting and broad coverage across prevalent CRKP lineages. Guided by recent epidemiological studies [33, 34], we selected representative strains: KL47 (334847), KL25 (1725‐2), KL20 (161), KL2 (8‐41), and KL112 (C7993), and evaluated the bactericidal activity of either single engineered phages or phage mixtures over 3 h. The results showed that RCIP0109*_R7_* effectively cleared the KL47–KL25 mixed culture across a range of multiplicity of infection (MOIs) (0.1–10) (Figure 5e; Figure S8a). Similarly, RCIP0109*_R5_* efficiently eliminated the KL2, KL20, and KL112 mixed culture at MOIs of 1–10 (Figure 5f; Figure S8b). When all five KL‐type strains were combined, RCIP0109*_R7_* or RCIP0109*_R5_* alone exhibited limited clearance, whereas the two‐phage cocktail achieved efficient eradication of the mixed bacterial community (Figure 5g; Figure S8c). These results demonstrate that rational phage cocktail design based on modular RBP engineering can provide predictable and comprehensive coverage against clinically diverse CRKP populations.

### 2.7 Assembly of Heterologous RBP2 on Phage RBP1 Generates Multivalent Phage Populations

**FIGURE 6:**
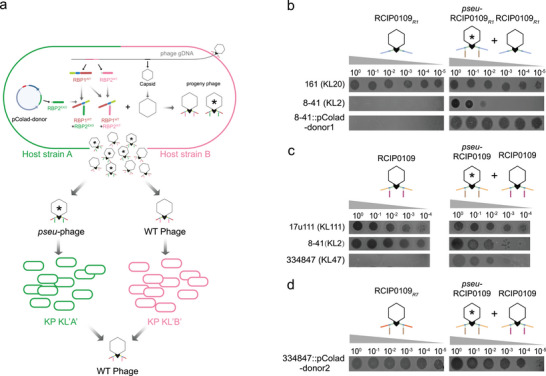
Modular incorporation of exogenous RBP2 diversifies the progeny phage population. (a) Schematic of the assembly workflow: during virion assembly in the host cell, phage particles can incorporate RBP2 proteins from different sources, yielding mixed progeny populations with distinct host ranges (*pseu*‐RCIP0109 denotes pseudotyped RCIP0109). (b) Compared with RCIP0109*_R1_*, the mixed‐state pseu‐RCIP0109*_R1_* and RCIP0109*_R1_* additionally lyse KL2 hosts, and can generate replicative progeny on KL47 hosts that supply exogenous RBP2. (c) Compared with RCIP0109, the mixed‐state *pseu*‐RCIP0109 and RCIP0109 additionally lyse KL2 hosts, and can generate replicative progeny on KL2 hosts that supply exogenous RBP2. (d) the mixed‐state *pseu‐*RCIP0109 and RCIP0109 exhibits plaque‐forming and replication efficiency on the relevant host comparable to that of the genomically engineered phage RCIP0109*_R7_*.

Phages are highly modular nanoscale machines whose tail structures typically assemble independently. Given that the combinatorial pairing of Prz_RBP1 and Prz_RBP2 modules enables programmable phage tropism, we hypothesized that providing multiple Prz_RBP2 variants within bacteria would allow their co‐assembly with Prz_RBP1, thereby generating phage populations with diverse Prz_RBP1‐RBP2 combinations. To test this hypothesis, we designed an experiment in which an exogenous Prz_RBP2 is expressed from a plasmid within the host cell, enabling phages to incorporate both native and exogenous RBP2 during infection and assembly. This approach is expected to produce progeny phages with an expanded host range (Figure 6a).

We constructed an arabinose‐inducible plasmid, pColad‐donor1, carrying the RCIP0109_RBP2 module targeting KL2, and transformed it into strain 161 (KL20). When phage RCIP0109*_R1_* (which encodes only Prz_RBP1 targeting KL20) infected strain 161 harboring this plasmid, the resulting progeny phages exhibited an expanded host range (Figure 6b). The progeny phages formed plaques on the KL20 strain, indicating normal infection by RCIP0109*_R1_*. On the KL2 host, high‐density spot assays revealed a characteristic lysis‐from‐without (LO) phenotype, producing localized cell death without discrete plaque formation. These results indicated that the progeny population comprised the parental RCIP0109*_R1_* and an engineered variant designated pseudo‐RCIP0109*_R1_* (pseu‐RCIP0109*_R1_*), which assembled both RBP1 and the exogenous RBP2 while retaining the RCIP0109R1 genome. In addition, after passaging these progeny phages in the plasmid‐harboring strain 8–41 (expressing the KL2 receptor), it was found that the engineered variant pseu‐RCIP0109*_R1_* could stably propagate in this host background.

Then, we infected the 17u111 strain harboring plasmid pColad‐donor2 encoding P560_RBP2 (targeting KL47) with phage RCIP0109, which naturally carries Prz_RBP1 and Prz_RBP2. The resulting progeny phages formed plaques on both KL111 and KL2 strains. Notably, high‐density spot assays on KL47 plates revealed a LO phenotype (Figure 6c). These progeny phages could stably propagate in the plasmid‐harboring strain 334847 (expressing the KL47 receptor), and their titers were comparable to those of the synthetic phage RCIP0109*_R7_* that specifically targets KL47 (Figure 6d).

In summary, these results confirm that even in the presence of the native RBP2, exogenously supplied RBP2 variants can efficiently integrate into assembling phage particles by pairing with the host phage's intrinsic Prz_RBP1. This assembly strategy can generate mixed phage populations with significantly expanded host ranges, directly verifying our proposed hypothesis of modular random assembly of RBPs.

## 3 Discussion

In this study, we demonstrate a complete workflow that bridges the gap between large‐scale phage genomic resources and functionally validated, customizable phage therapeutics against multidrug‐resistant pathogens. We selected *K. pneumoniae* as a clinically relevant model due to its extensive capsular diversity and its status as a WHO‐designated priority pathogen [17, 35]. The capsule of *K. pneumoniae*, while essential for phage recognition, is highly diverse and thus presents a significant obstacle to effective phage therapy, as most phages recognize only a single capsule type [33, 36]. This high antigenic diversity necessitates a correspondingly diverse set of RBPs, which are central to host recognition and capsule degradation [29, 31]. However, despite the rapid accumulation of RBP sequences in public databases, only a small fraction has been functionally annotated, and the modular principles that govern their host specificity remain poorly defined.

To address this, we focused on the genus *Przondovirus*, whose phages possess the largest known genomic representation among *Klebsiella*‐infecting phages and collectively target the broadest KL spectrum. Comparative analysis of 280 *Przondovirus* RBPs revealed multiple well‐defined sequence clusters: amino‐acid identity is high within each cluster but drops sharply between neighboring clusters. Fourteen clusters already contain biochemically verified RBPs that target distinct K‐antigen (capsule) types (Table S1), underscoring a tight link between RBP sequence and receptor tropism. The many clusters that remain unannotated, therefore constitute a rich reservoir of putative RBPs whose specificities are still unknown and serve as the starting point for our study.

Building on this observation, we devised a bottom‐up workflow that combines in‐silico clustering with experimental screening to systematically assign function to *Przondovirus* RBPs. Functional validation of 41 previously uncharacterized RBPs expanded the known targeting spectrum to 32 capsule (KL) types, several of which dominate today's multidrug‐resistant *K. pneumoniae* lineages. A parallel survey of *Staphylococcus aureus* phages demonstrated the generality of our approach: distinct RBP clusters there exhibit specific preferences for particular wall‐teichoic‐acid backbones or glycosylation variants, confirming that cluster‐based classification captures biologically meaningful receptor specificity across bacterial species [37]. Interestingly, KL57‐targeting RBPs were found in both Prz_RBP1 and Prz_RBP2 clades, sharing up to 90% identity in conserved catalytic domains (Figure S1b), suggesting that RBP modularity may promote horizontal gene transfer among phages [28, 38]. In addition, we observed that some RBPs can target multiple KL types. For instance, among the three RBP members in the KL111 cluster, although their amino acid sequences share over 90% identity and their structures are nearly identical, NC_047968_RBP1 and RCIP0109_RBP1 can still additionally recognize KL22 and KL13, respectively. This may result from subtle changes in the RBPs’ capsule‐binding sites that increase their tolerance, enabling recognition of other KL types. Such cross‐KL recognition has also been reported in previous studies [14, 39]. Overall, these observations suggest that minor sequence variations within RBPs can confer additional KL‐specific recognition.

We systematically analyzed the RBPs from 280 *Przondovirus* phages and found that, based on domain architecture, they can be categorized into two distinct classes, which we designate as Prz_RBP1 and Prz_RBP2. Eighty‐four Prz_RBP1 proteins harbor a T4gp10‐like branching domain, whereas every Prz_RBP2 contains a ∼30‐aa conserved peptide that fulfills an analogous anchoring function. In previous studies, structural and biochemical evidence indicated that the T4gp10‐like domain and these conserved peptides may assemble two or more tailspikes in a branched configuration [32, 40]. Because these scaffolds are pervasive throughout the *Przondovirus* genus, they may provide a ready‐made genetic toolbox for modular construction of engineered phages targeting multiple capsular types. We demonstrated that Prz_RBP1, which harbors a T4gp10‐like domain, and Prz_RBP2, which contains a conserved N‐terminal motif, function as modular and combinable units. Recombinant phages assembled with diverse Prz_RBP1 and Prz_RBP2 proteins from different phages exhibited predictable shifts in host range, corresponding to the specificity of the exchanged RBPs. By exploiting the ability of Prz_RBP1 and Prz_RBP2 to pair freely, we developed a strategy that enables the construction of a phage population capable of targeting multiple capsular types. We supplied the Prz_RBP2 gene exogenously, allowing it to be seamlessly incorporated into assembling virions. This modular compatibility effectively circumvents genome packaging constraints and eliminates the need for complex genetic reconstruction, thereby enabling the generation of progeny phages with broadened host ranges at minimal engineering cost.

Taken together, our study presents a scalable, system‐level strategy that transforms available phage genomic data into a functional repository of modular parts. This enables the construction of programmable phages with tailored host specificity, overcoming current limitations in phage therapy and setting the stage for precision antimicrobials built on a synthetic biology foundation.

## 4 Methods

### 4.1 Bacterial Strains and Growth Conditions

Unless otherwise specified, all bacterial strains, phages, and plasmids used in this study are listed in Table S2. *K. pneumoniae* and *Escherichia coli* strains were routinely cultured in Luria‐Bertani (LB) medium (Oxoid, CM0996B), either as liquid broth or on agar plates containing 15 g/L agar (AMEKO). Cultures were typically incubated at 37°C with shaking at 220 rpm. When appropriate, antibiotics were supplemented as follows: kanamycin at a final concentration of 50 µg/mL and gentamicin at 20 µg/mL.

### 4.2 Phage Propagation

Overnight cultures of phage‐specific propagation hosts were subcultured in fresh medium and grown to mid‐logarithmic phase (approximately ∼10^8^ CFU/mL). Phage stock (approximately ∼10^7^ PFU/mL) was added and incubated at 37°C for 3–5 h or visible lysis was observed. The lysate was centrifuged (10 000 ×g, 15 min), and the resulting supernatant was passed through a 0.22 µm filter and stored at 4°C.

### 4.3 Phage DNA Isolation

Phages were propagated in liquid cultures using their respective host strains. Following incubation, cultures were treated with chloroform to lyse bacterial cells. The resulting lysates were clarified by filtration through 0.22 µm membrane filters. Phage genomic DNA was then extracted using the Lambda Phage Genomic DNA Extraction Kit (ZOMANBIO, Beijing, China), according to the manufacturer's instructions.

### 4.4 Phage Titer Assay

Phage titer was determined by the double‐layer agar plaque assay [41]. Briefly, 100 µL of serially diluted phage was mixed with 100 µL of mid‐log‐phase host bacteria (OD_600_ = 0.6) and 5 mL of 0.6% molten soft agar. The mixture was gently mixed by inversion and poured onto solidified LB agar plates. After overnight incubation at 37°C, plaques were enumerated. PFU totals between 10 and 300 PFU were typically considered acceptable, otherwise plating was repeated for the same dilution series. Each sample was measured in three independent technical replicates, and the average value was reported as the final titer.

### 4.5 *Klebsiella* Phage Dataset Construction

The *Klebsiella* phage dataset was constructed by retrieving all records containing the keyword “*Klebsiella* phage” from the NCBI GenBank database (as of March, 2025). Only records annotated as complete genomes were retained to exclude incomplete or fragmented sequences. In addition, phages isolated from non‐*Klebsiella* hosts were removed based on host information.

### 4.6 Phage Annotation and RBP Identification

To achieve standardized annotation of phage genomes and retrieve genomic information from unannotated entries, we first performed genome reordering on all phage sequences retrieved from public databases using the one‐stop phage analysis pipeline (https://nmdc.cn/phage/tools/phage). Genome annotation was performed as described previously [14]. Briefly, open reading frames (ORFs) were predicted and annotated using Prokka (v1.14.6) [42] with the parameters –kingdom Viruses and –metagenome. The resulting protein sequences were further annotated using multiple approaches: (i) submission to the NCBI Batch CD‐Search web server with Pfam and TIGR databases specified [43]; (ii) submission to the KofamKOALA web server based on the Kyoto Encyclopedia of Genes and Genomes (KEGG) database [44]; and (iii) BLASTp search against sequences from VOGDB (https://vogdb.org/) using identity >40% and coverage >40% as thresholds. Phages with annotated RBPs (tail fibers) were directly processed for RBP extraction. For phages in which RBPs could not be identified through annotation, genome sequence alignment was performed using Easyfig to confirm RBP loci before extraction [45]. All extracted RBP sequences were further annotated using HHpred (https://toolkit.tuebingen.mpg.de/tools/hhpred) [46] to validate the reliability of RBP identification.

### 4.7 RBP Clustering and Phylogenetic Analysis

Given that even minor amino acid substitutions can affect RBP host specificity, CD‐HIT was applied at a 100% sequence identity threshold to eliminate redundancy [47, 48]. Multiple sequence alignment of the RBPs was performed using MAFFT [49]. A phylogenetic tree was subsequently constructed and visualized with the Interactive Tree of Life (iTOL) [50] to assess the evolutionary relationships among the RBP sequences. Node stability was quantified using ultrafast bootstrap approximation (UFBoot, 1000 replicates), and the resulting support values were shown next to the corresponding branches.

### 4.8 Cloning, Expression, and Purification of Phage RBPs

RBP genes selected from our previously constructed phage library were obtained by PCR amplification of purified phage genomic DNA, while RBP genes retrieved from public databases were synthesized by Sangon Biotech (Sangon Biotech, Shanghai, China) and directly cloned into the pET28a plasmid. All RBP sequences were directionally cloned into the pET‐28a(+) vector between the *Nde* I and *BamH* I restriction sites using Gibson assembly [51]. The assembled recombinant plasmids were first transformed into *E. coli* Top10 for propagation and plasmid extraction, and then introduced into *E. coli* BL21(DE3) via heat shock for protein expression. Primer sequences and synthetic gene fragments used for cloning are listed in Table S3.

For overexpression, *E. coli* BL21(DE3) cells harboring the RBP expression plasmid were cultured overnight at 37°C with shaking (220 rpm), and subcultured into fresh LB medium containing 50 µg/mL kanamycin. At an OD_600_ of ∼0.6, protein expression was induced with 0.5 mM IPTG, followed by incubation at 18°C for 14 to 16 h (150 rpm). Cells were harvested by centrifugation at 5,000 × g for 10 min, washed with PBS, and resuspended in lysis buffer (50 mm Tris‐HCl, 200 mm NaCl, 5 mm imidazole, pH 8.0). After sonication and clarification (10,000 × g, 30 min), the supernatant was loaded onto a Ni‐NTA affinity column to purify His‐tagged RBP proteins. Eluted proteins were assessed by SDS‐PAGE, and high‐purity fractions were concentrated using 30 kDa MWCO centrifugal filters (Millipore).

### 4.9 RBP Host Tropism (Spotting)

Purified RBPs (*n* = 41) were evaluated for depolymerase activity and host range specificity against a panel of 174 *Klebsiella* strains representing diverse capsular types (KLs) using a spot assay. Before performing the spot assay, the purified RBPs were adjusted to a stock concentration of 1 mg/mL and serially diluted with PBS to final concentrations of 100, 10, and 1 ng/µL. For each dilution, 5 µL was spotted onto LB agar plates previously overlaid with a lawn of the respective bacterial strain. After air‐drying, plates were incubated at 37°C, and the presence of lysis or halo zones indicating depolymerase activity was recorded at 12 h post‐incubation. All assays were performed in triplicate to ensure reproducibility.

### 4.10 Structural Alignment

The structure of each RBP was predicted using AlphaFold3 (https://alphafoldserver.com) [52] with the full‐length protein sequences as input, and visualized in PyMOL (v2.5.8). Structural comparisons between RBPs were performed using Foldseek (https://search.foldseek.com/) [53], and MSA LDDT (Local Distance Difference Test) scores were reported as the output metric. For structural alignment of the T4gp10‐like domain, residues 188–256 of RCIP0109_RBP1 (WPJ56834.1), residues 185–256 of RCIP0018_RBP1 (WPJ49360.1), and residues 188–257 of Kp‐phage P560_RBP1 (QOV05501.1) were used.

### 4.11 Phage Engineering

Phage engineering as previously described [14]. Specifically, to construct the donor plasmid, primers were used to amplify the full‐length donor RBP as well as approximately 200 bp of homologous regions upstream and downstream of the chassis phage RBP. These overlapping fragments were then assembled into a linearized pCOLADRed vector via Gibson assembly. The positions of the homologous fragments were adjusted according to design requirements. Correctly assembled donor plasmids were verified by sequencing and subsequently transformed into the Kp8‐41 host strain for downstream phage recombination experiments.

During the recombination experiments, the recombinase system in Kp8‐41 carrying the donor plasmid was induced with 1 mm arabinose. Once the bacteria reached logarithmic growth, wild‐type phage RCIP0109 was added and co‐cultured at 37°C for 30–60 min. Chloroform was then added and the culture vigorously mixed to lyse the cells and release the phage. The resulting phage lysate was plated with the corresponding host using the double‐layer agar method to select for successfully recombined phages. Positive plaques were subjected to at least three rounds of infection and screening to eliminate wild‐type phage background and stabilize the phage phenotype.

The construction of the *pseu*‐phage was performed as follows: The exogenous RBP2 fragment was amplified using specific primers with overlapping sequences, while the pCOLADRed plasmid was linearized and its original λERD (Exo, Beta, Gam) region was removed through primer design. This allowed the exogenous RBP2 to be inserted at the same locus and placed under arabinose‐inducible control. The fragments were then assembled using Gibson Assembly, and the resulting recombinant plasmid was transformed into *E. coli* Top10 competent cells. After sequence verification, the correctly assembled pColad‐donor plasmid was introduced into the target host strain. Cells carrying this donor plasmid were induced with 1 mm arabinose to express the exogenous RBP2 and subsequently infected by phage during the logarithmic growth phase, resulting in the production of both natural phage progeny and *pseu*‐phage within the host. The primers used are listed in Table S3.

### 4.12 Kinetic Curves of Phage Killing

Overnight cultures of various *K. pneumoniae* serotype strains (KL20: 161, KL47: 334847, KL25: 1725‐2, KL2: 8–41, KL112: C7993) were subcultured by 1:200 dilution into fresh medium. Once the cultures reached an optical density at 600 nm of 0.2, a 20 µL aliquot from each of the five bacterial cultures was pooled, resulting in a final concentration equivalent to 10^7^ CFU. This mixture was then transferred to a 100‐well plate (Honeycomb Microplate). Predetermined titers of the phage cocktails or individual phages were then added to the corresponding wells to achieve the target (MOI). The composition of the bacterial types within different groups was adjusted according to specific experimental requirements, while ensuring the total bacterial load remained constant. Finally, the growth kinetics were determined using an automated growth curve analyzer (Bioscreen C), with OD_600_ measurements recorded every 15 min. All growth curves were corrected by subtracting the background signal (LB medium containing SM buffer).

The mixtures were prepared in the same ratio in test tubes, and bacteria were collected after 3 h. The cells were washed three times with PBS, serially diluted, plated on LB agar, and incubated at 37°C. Colony counts were used to determine CFU/mL. All experiments were performed in three independent biological replicates. Statistical analyses were performed using GraphPad Prism v.9.1. CFU counts between phage‐treated and control groups were compared using a two‐tailed Mann–Whitney *U test* (unpaired). Data are presented as boxplots showing median and interquartile range, with whiskers representing minimum to maximum values, and individual data points overlaid. *p* < 0.05 was considered statistically significant.

### 4.13 Phage Host Range Determination

The host range of wild‐type and engineered phages was assessed by spot assays [54]. Briefly, 200 µL of mid‐log‐phase *K. pneumoniae* culture was mixed with 5 mL of 0.6% molten soft agar and overlaid onto LB agar plates to form a bacterial lawn. Subsequently, 5 µL of serially diluted phage preparations were spotted onto the surface. Plates were incubated overnight at 37°C, and plaque formation was examined the following day. All assays were performed in triplicate to ensure reproducibility.

### 4.14 Extraction and Digestion of CPS

*K. pneumoniae* strains were grown overnight in LB broth at 37°C with shaking at 200 rpm. CPS were extracted using a commercial CPS Extraction Kit (EX1750, Solarbio Life Sciences, Beijing, China), following the manufacturer's instructions. Extracted CPS samples were separated on 6% SDS‐PAGE gels and visualized using Alcian blue staining [55]. Briefly, the gels were fixed in a solution containing 25% ethanol and 10% acetic acid (v/v) at 50°C for three washes of 10 min each, followed by staining in 0.125% Alcian blue solution under light‐protected conditions at 50°C for 15 min. Gels were then destained at room temperature using the same fixing solution until distinct bands were visible against a light background.

To evaluate the depolymerization activity of RBPs, CPS extracted from 4 mL of bacterial culture was incubated with either 2 µg of purified RBP or an equal volume of PBS at 37°C for 5 h. The digestion products were then analyzed by SDS‐PAGE to assess CPS degradation.

### 4.15 Construction of Capsule‐Deficient *K. pneumoniae* Mutants

All gene knockout mutants were constructed using a CRISPR‐Cas9 system in combination with λ‐Red‐mediated homologous recombination [56]. This system consisted of two plasmids: pCOLADRed, which confers kanamycin resistance and expresses λ‐Red recombinases to enhance homologous recombination efficiency; and pCas9gRNA, which confers gentamicin resistance and expresses Cas9 along with gene‐specific sgRNAs to eliminate non‐recombined wild‐type cells. Briefly, *K. pneumoniae* strains were first transformed with pCOLADRed via electroporation, and λ‐Red expression was induced with 1% (w/v) L‐arabinose. Subsequently, cells harboring pCOLADRed were co‐electroporated with the pCas9gRNA plasmid and a repair template containing homologous arms.

Transformants were selected on LB agar plates supplemented with 20 µg/mL gentamicin and 50 µg/mL kanamycin. Successful gene deletions were confirmed by PCR and Sanger sequencing. For KL25 and KL64 strains, sgRNAs were designed to target the *wcaJ* gene; for KL20, KL107, and KL112 strains, sgRNAs targeted the *wbaP* gene. The primers used are listed in Table S3.

### 4.16 Statistical Analysis

Statistical analyses were performed using GraphPad Prism v9.1. Depending on the experimental design, mean ± SEM or median values were calculated, and the appropriate statistical tests were applied. The specific tests used are indicated in the corresponding Methods sections.

## Author Contributions

J.S.S.: Conceptualization, Data curation, Formal analysis, Methodology, Validation, Visualization, Writing – original draft and review. S.Y.Y.: Data curation, Formal analysis, Methodology, Validation, Visualization. B.X.B.: Data curation, Formal analysis, Methodology, Validation. S.Y.Q.: Formal analysis, Methodology, Software. W.D.W.: Formal analysis, Project administration, Supervision. H.J.Q.: Data curation, Formal analysis, Investigation, Methodology. Z.Y.: Software, Supervision. Z.G.: Investigation, Methodology, Project administration. Z.R.: Resources. W.C.: Conceptualization, Formal analysis, Supervision, Writing – review & editing. F.J.: Conceptualization, Formal analysis, Funding acquisition, Resource, Writing – original draft: Lead; Writing – review & editing.

## Conflicts of Interest

The authors declare no conflicts of interest.

## Supporting information




**Supporting File 1**: advs74222‐sup‐0001‐SuppMat.pdf.

**Supporting File 2**: advs74222‐sup‐0002‐TableS1.xlsx.

**Supporting File 3**: advs74222‐sup‐0003‐TableS2.xlsx.

**Supporting File 4**: advs74222‐sup‐0004‐TableS3.xlsx.

## Data Availability

The genome sequences of phages RCIP0133, RCIP0140, RCIP0141, RCIP0152, RCIP0153, RCIP0154, RCIP0157, RCIP0165, RCIP0174, RCIP0210, RCIP0213, RCIP0215, RCIP0226, RCIP0229, and RCIP0234 have been deposited in the NCBI GenBank database under the accession numbers PV854167–PV854181, respectively. All protein sequences analyzed in this study are publicly available from the NCBI database with accession numbers: RCIP0018_RBP1(WPJ49360.1), RCIP0003_RBP2(WPJ47567.1), RCIP0069_RBP2(WPJ53906.1), RCIP0109_RBP1(WPJ56834.1), RCIP0109_RBP2(WPJ56835.1), RCIP0101_RBP1(WPJ56106.1), RCIP0101_RBP2(WPJ56107.1), RCIP0060_RBP2(WPJ53315.1), vB_KpnP_IL33_RBP1(ARB12452.1), 066046_RBP1(QOV07395.1), KPN3_RBP1(QEG11145.1), VLCpiA3d_RBP1(UVX31290.1), BUCT‐3589_RBP1(UXQ90405.1), P560_RBP1(QOV05501.1), Pharr_RBP1(QBZ71248.1), vB_Kpn_K43PH164C1_RBP1(CAK6604344.1), KP32_isolate 194_RBP1(AWN07125.1), KP32_isolate 192_RBP1(AWN07083.1), vB_Ko_K26PH128C1_RBP1(CAK6597195.1), Whistle_RBP1(WEU80648.1), Henu1_RBP1(AZS06409.1), Kund‐ULIP54_RBP2(QBG78386.1), vB_KpnP_KpV766_RBP2(AOZ65570.1), vB_Ko_K74PH129C2_RBP2(CAK6597303.1), KP32_isolate 196_RBP2(AWN07214.1), Henu1_RBP2(AZS06408.1), 175009_RBP1(WWT41176.1), SH‐KP152226_RBP2(QDF14645.1), 175002_RBP1(WWT40676.1), vB_Kpl_K48PH164C1 RBP2(CAK6589138.1). The data that support the findings of this study are available from the corresponding author upon reasonable request.
