# Supplementary material for: Programmable Klebsiella pneumoniae Phage Tropism Enabled by Scalable Receptor‐Binding Protein Mining and Modular Assembly
Source: Adv Sci (Weinh). 2026 Feb 19;13(21):e14511. doi: 10.1002/advs.202514511 (PMC13073335; doi:10.1002/advs.202514511)
Supplement: Supplementary file 1 — Supporting File 1: advs74222‐sup‐0001‐SuppMat.pdf. [file ADVS-13-e14511-s004.pdf]

Programmable Klebsiella pneumoniae Phage Tropism Enabled by Scalable Receptor-Binding Protein Mining and Modular Assembly

Shisong Jing, Yiyao Song, Xianbiao Bi, Yuqin Song, Dawei Wei, Jiangqing Huang, Yuan Zeng, Gang Zhang, Rong Zhang, Chao Wang, Jie Feng

Fig. S1-8

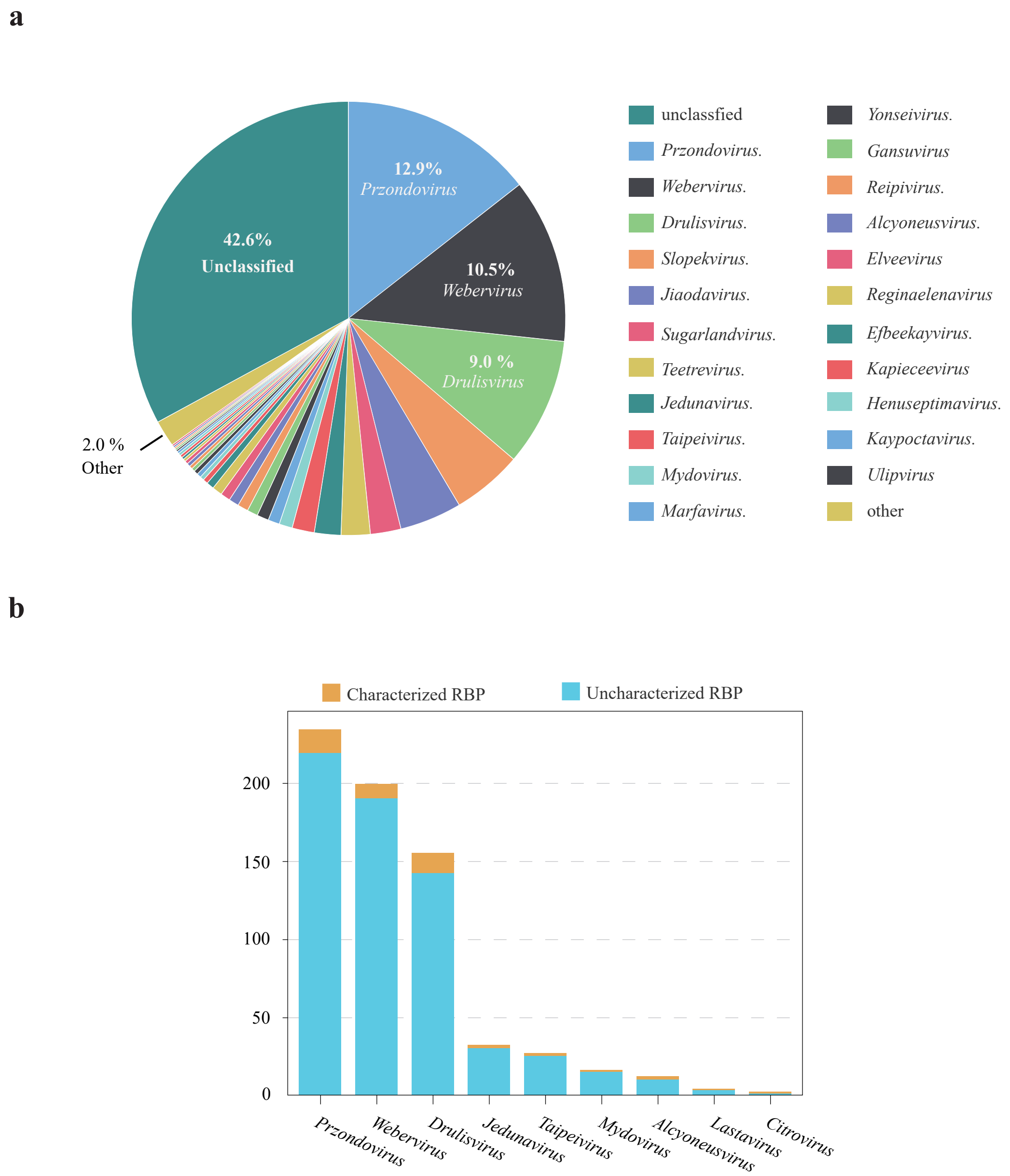

Supplementary Fig. 1. *Przondovirus* is the most abundant genus of *K. pneumoniae* phages, with a low proportion of characterized RBPs across genera supporting the representativeness of our analysis. (a) Pie chart showing the proportions of *K. pneumoniae* phages across different genera. Excluding unclassified phages, the three most abundant genera are *Przondovirus* (12.9%), *Webervirus* (10.5%), and *Drulisvirus* (9%). **(b)** Stacked bar chart showing the number of phages with characterized or uncharacterized capsule polysaccharide-targeting RBPs in each genus. Yellow indicates phages with characterized RBPs, and blue indicates phages without characterized RBPs. In all genera, only a small proportion of phages have characterized RBPs, demonstrating that no genus is disproportionately overrepresented in RBP characterization.

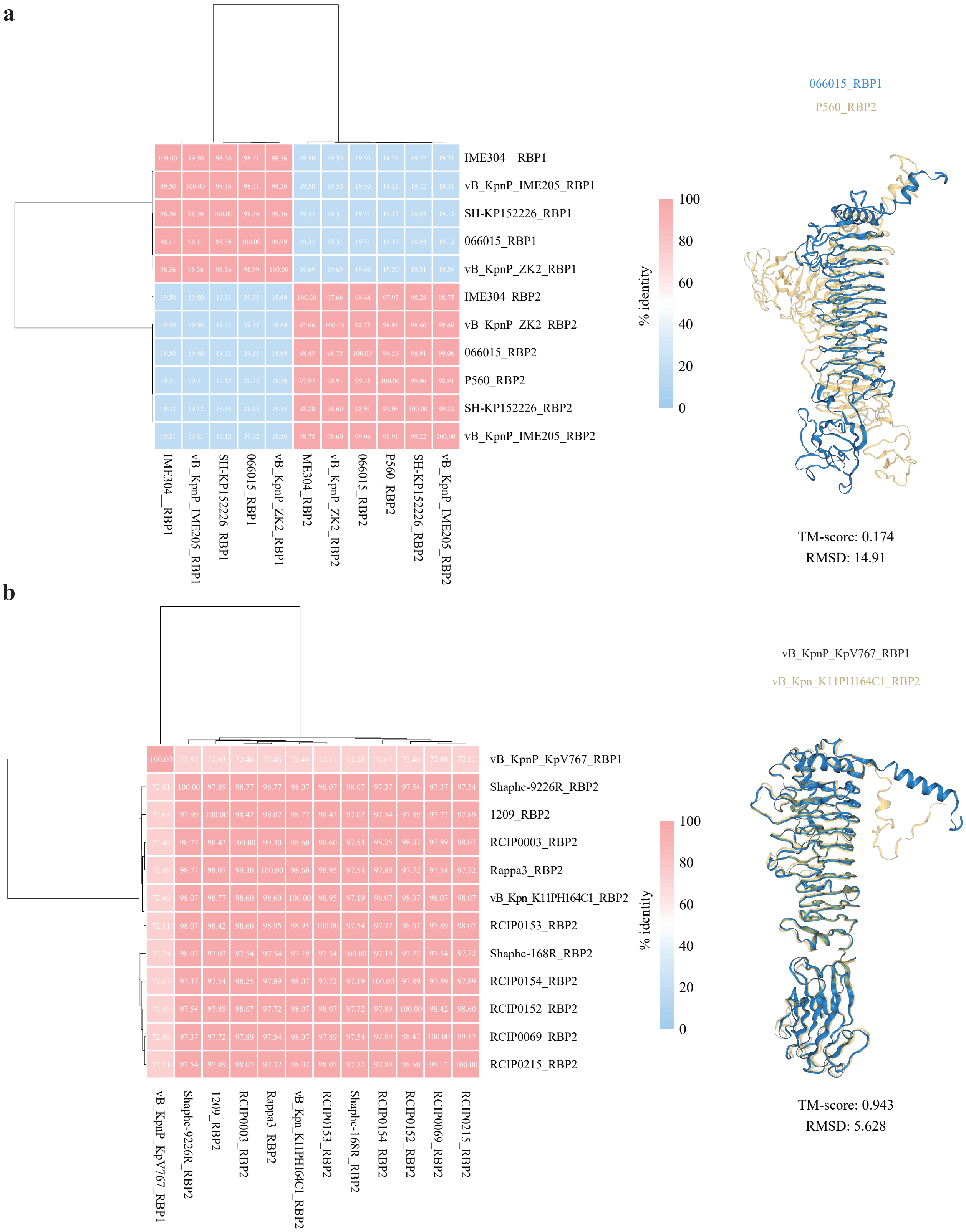

Supplementary Fig. 2. Sequence and structural comparison of Prz_RBP1 and Prz_RBP2 targeting KL47 and KL57. (a) No significant sequence similarity was observed between Prz_RBP1 and Prz_RBP2 targeting KL47. Structural alignment using Foldseek revealed no structural similarity between the representative proteins 066015_RBP1 and P560_RBP2. **(b)** In contrast, the catalytic domains of RBPs targeting KL57 showed high sequence conservation between Prz_RBP1 and Prz_RBP2, suggesting that this domain may have undergone horizontal transfer through a modular mechanism. Structural alignment using Foldseek demonstrated high structural similarity between the representative proteins vB_KpnP_KpV767_RBP1 and vB_Kpn_K11PH164C1_RBP2.

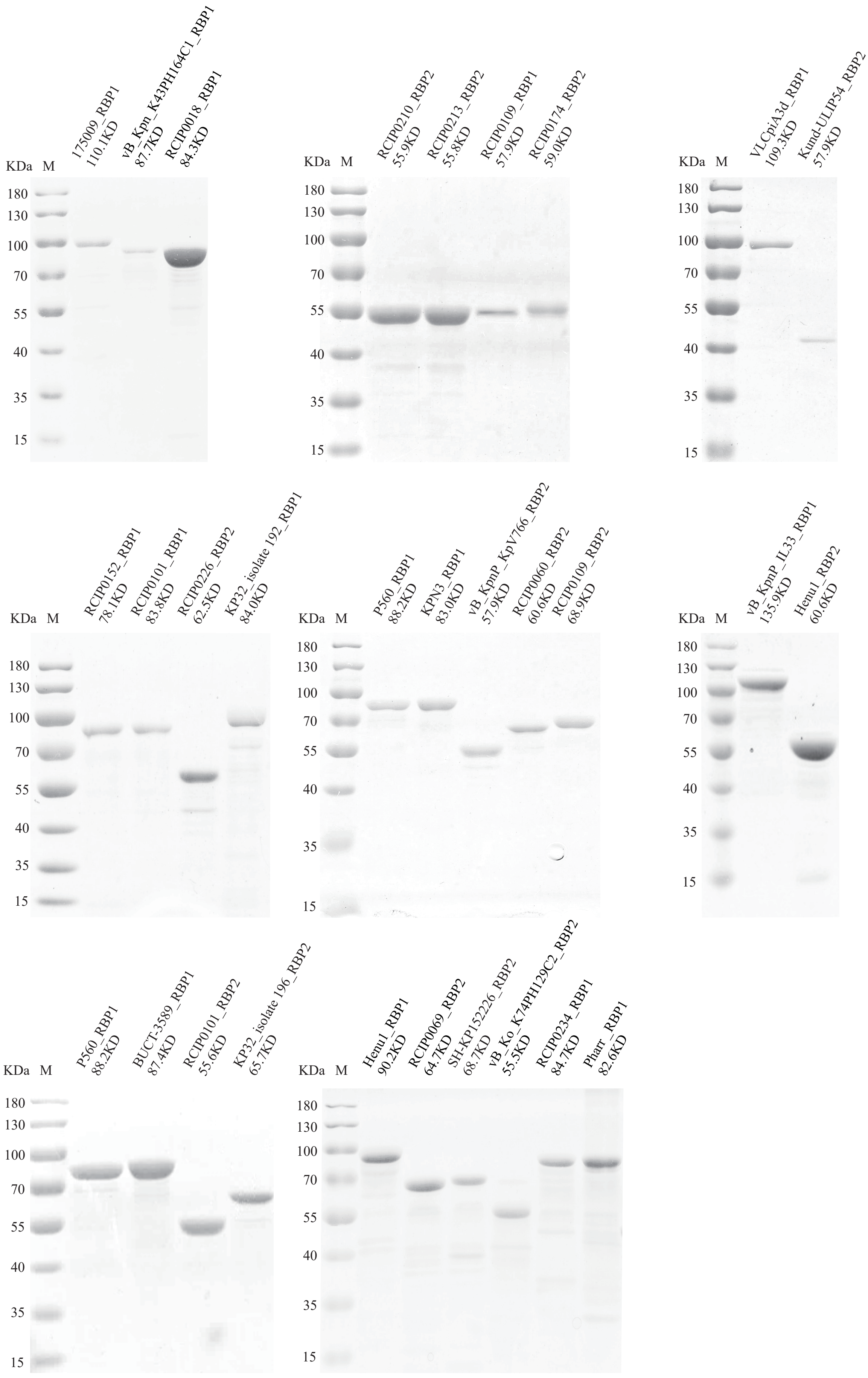

KDa M
175009_RBP1 110.1KD
vB_Kpn_K43PH164C1_RBP1 87.7KD
RCIP0018_RBP1 84.3KD
180
130
100
70
55
40
35
15
KDa M
RCIP0210_RBP2 55.9KD
RCIP0213_RBP2 55.8KD
RCIP0109_RBP1 57.9KD
RCIP0174_RBP2 59.0KD
KDa M
VLCpiA3d_RBP1 109.3KD
Kund-ULIP54_RBP2 57.9KD
KDa M
RCIP0152_RBP1 78.1KD
RCIP0101_RBP1 83.8KD
RCIP0226_RBP2 62.5KD
KP32_isolate 192_RBP1 84.0KD
KDa M
P560_RBP1 88.2KD
KPN3_RBP1 83.0KD
vB_KpnP_KpV766_RBP2 57.9KD
RCIP0060_RBP2 60.6KD
RCIP0109_RBP2 68.9KD
KDa M
vB_KpnP_IL33_RBP1 135.9KD
Henu1_RBP2 60.6KD
KDa M
P560_RBP1 88.2KD
BUCT-3589_RBP1 87.4KD
RCIP0101_RBP2 55.6KD
KP32_isolate 196_RBP2 65.7KD
KDa M
Henu1_RBP1 90.2KD
RCIP0069_RBP2 64.7KD
SH-KP152226_RBP2 68.7KD
vB_Ko_K74PH129C2_RBP2 55.5KD
RCIP0234_RBP1 84.7KD
Pharr_RBP1 82.6KD

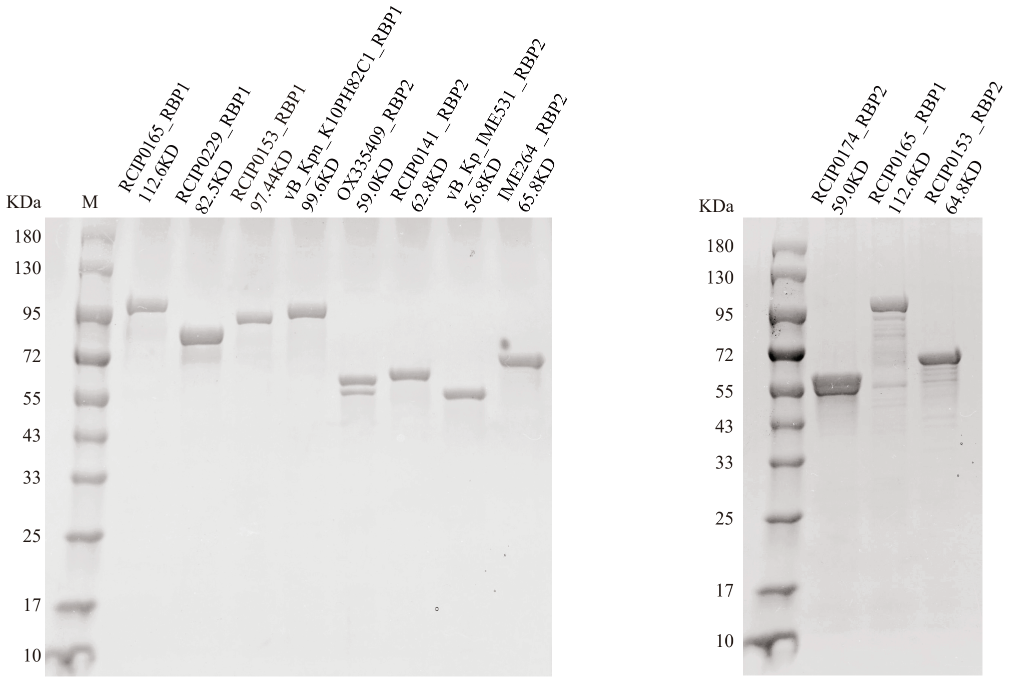

Supplementary Fig. 3. SDS-PAGE analysis of solubly expressed RBP proteins. A total of 41 RBP proteins with soluble expression were analyzed. The gel shows the migration bands corresponding to each protein and their respective molecular weights.

a

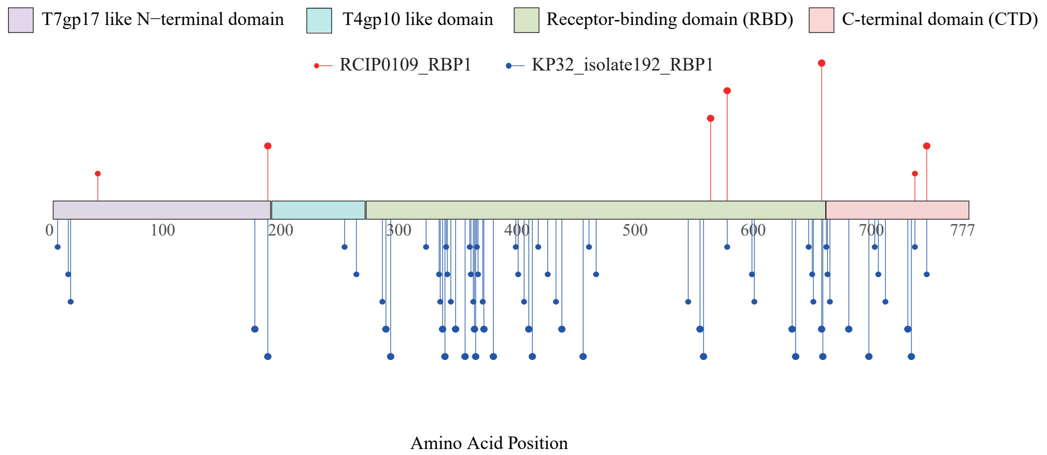

b

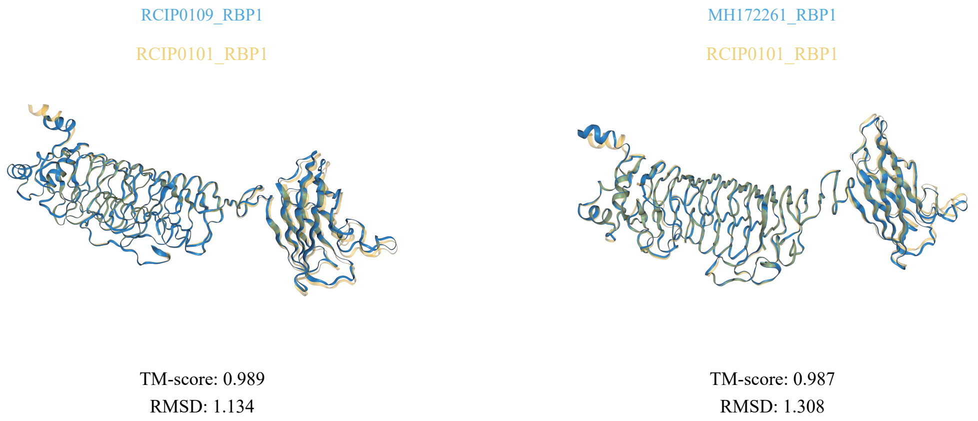

Supplementary Fig. 4. Sequence variation in RBPs drives changes in host range. (a) Lollipop plots show amino acid differences within the same RBP cluster between KP32_isolate192_RBP1 (targeting KL111 and KL22) or RCIP0109_RBP1 (targeting KL111 and KL13) and RCIP0101_RBP1 (targeting only KL111). The red and blue lollipops indicating positions of amino acid differences in RCIP0109_RBP1 and KP32_isolate192_RBP1, respectively. **(b)** Structural alignment of the proteins (amino acids 280–777) generated using Foldseek. Despite minimal sequence variation and high structural similarity, these localized differences appear sufficient to alter the host range of the RBPs.

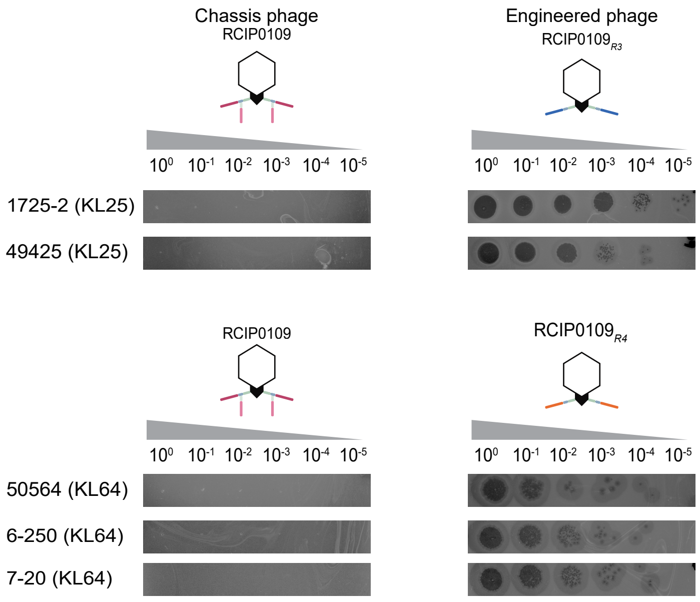

Supplementary Fig. 6. Reprogramming host specificity of the chassis phage through modular RBP replacement. Engineered phages RCIP0109$_{R3}$ and RCIP0109$_{R4}$ were able to lyse multiple tested strains of KL25 and KL64, respectively, demonstrating that switching the RBP successfully altered the host range of the phages.

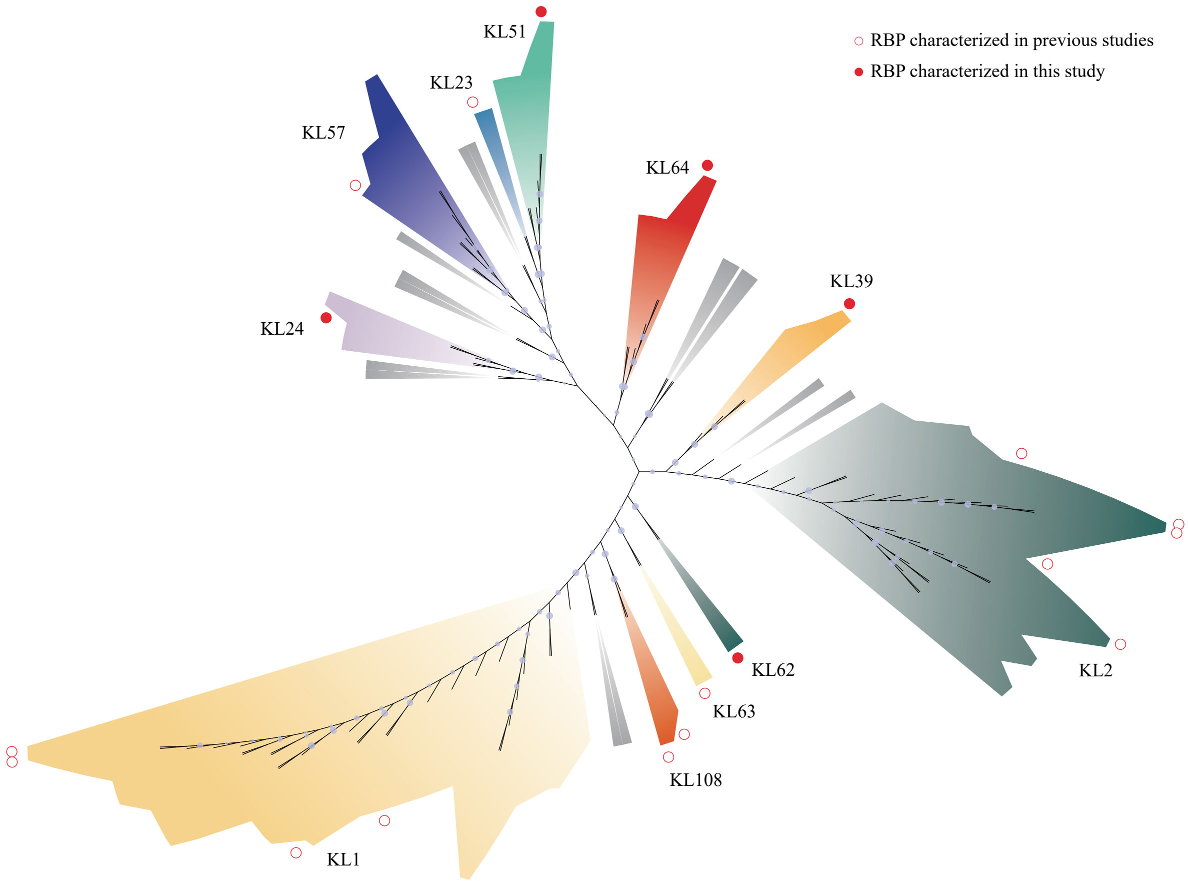

Supplementary Fig 5. Phylogenetic analysis of Dru_RBP2 reveals distinct RBP clusters corresponding to different *K. pneumoniae* capsular (KL) types. A maximum likelihood tree was constructed using 106 non-redundant Dru_RBP2 sequences from 155 *Drulivirus* phages. Most phages carried a single RBP, classified as Dru_RBP1 (65 sequences) or Dru_RBP2 (110 sequences), while 23 phages carried two RBPs. Dru_RBP2 sequences formed 27 distinct clusters, with the largest containing 29 members and the smallest a single member. Branch colors indicate associated KL types (gray, unknown KL). Previously characterized Dru_RBPs are shown as red open circles; RBPs selected and characterized in this study are shown as red filled circles. Spot assays confirmed that representative RBPs from five uncharacterized clusters—MZ634340_RBP2, MK135468_RBP2, PP974225_RBP2, OY978782_RBP2, and KP708985_RBP2—target KL51, KL24, KL64, KL39, and KL62, respectively, expanding the Dru_RBP component library. These results support a one-to-one correspondence between RBP clusters and KL types and demonstrate that this clustering framework can be generalized to other phage genera.

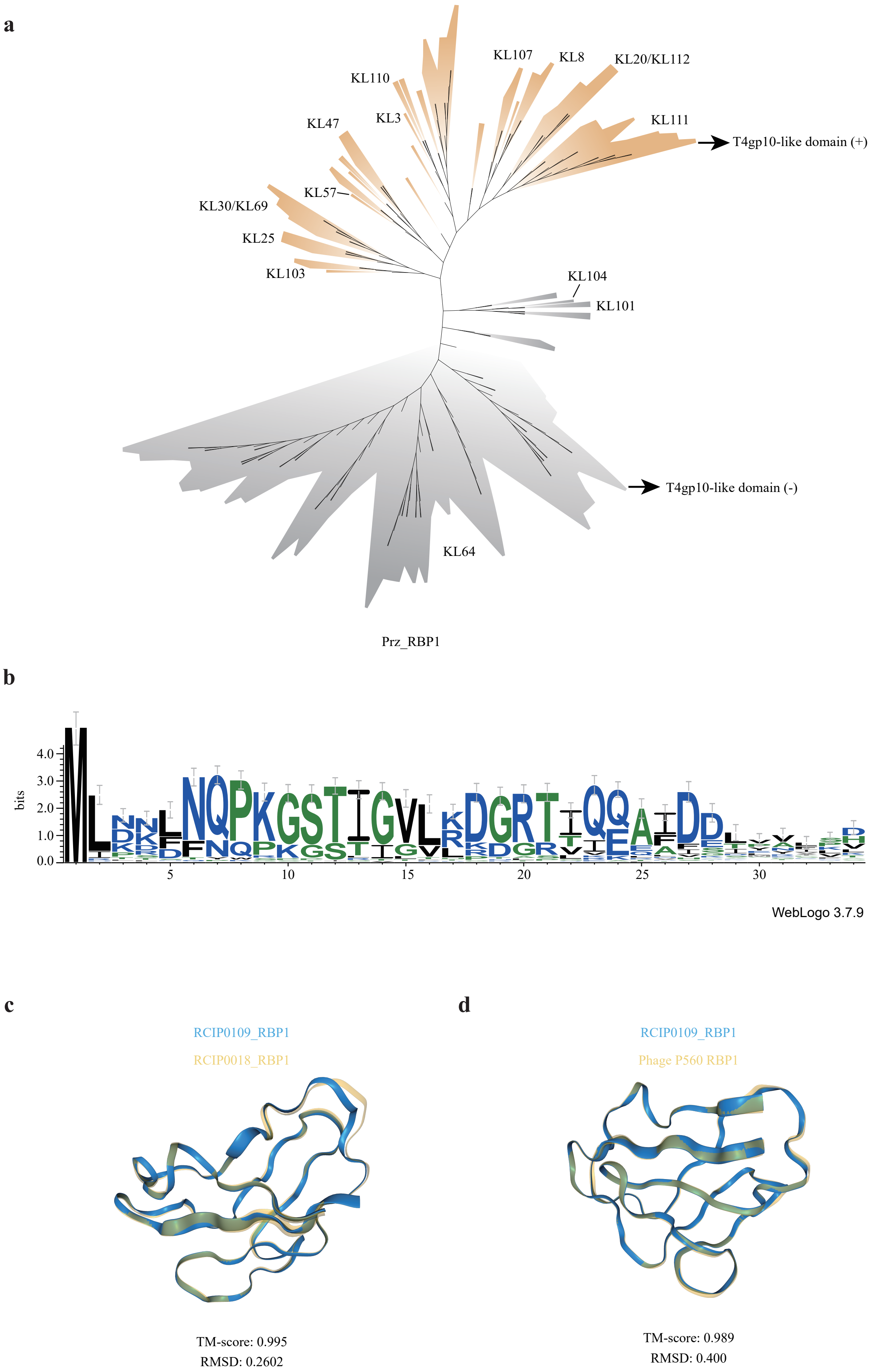
a
KL110
KL107
KL8
KL20/KL112
KL3
KL47
KL111
T4gp10-like domain (+)
KL57
KL30/KL69
KL25
KL103
KL104
KL101
T4gp10-like domain (-)
KL64
Prz_RBP1
b
bits
4.0
3.0
2.0
1.0
0.0
5
10
15
20
25
30
WebLogo 3.7.9
c
RCIP0109_RBP1
RCIP0018_RBP1
TM-score: 0.995
RMSD: 0.2602
d
RCIP0109_RBP1
Phage P560 RBP1
TM-score: 0.989
RMSD: 0.400

Supplementary Fig. 7. Conservation analysis of branching structures in Prz_RBPs. (a) Distribution of the T4gp10-like domain among 183 Prz_RBP1 proteins. Clusters with a yellow background indicate the presence of the T4gp10-like domain, while clusters with a gray background indicate its absence. **(b)** Sequence conservation of the N-terminal domains of all Prz_RBP2 proteins was analyzed using WebLogo 3.7.9. A highly conserved ~30-amino-acid motif was identified at the N-terminus of all Prz_RBP2 proteins, which is responsible for interaction with Prz_RBP1. **(c)** and **(d)** Structural alignments of the T4gp10-like domains were performed using Foldseek. The T4gp10-like domain of the chassis phage RCIP0109_RBP1 (amino acids 188–256) was compared with that of phage RCIP0018_RBP1 (amino acids 188–256) and Kp phage P560_RBP1 (amino acids 187–257), respectively. The TM-scores were 0.995 and 0.989, and the corresponding Root Mean Square Deviation (RMSD) values were 0.26 and 0.40, indicating a high degree of structural similarity between these domains. In the structural alignments, RCIP0109_RBP1 T4gp10 like domain is shown in blue, while RCIP0018_RBP1 T4gp10-like domain (panel c) and Phage P560_RBP1 T4gp10-like domain (panel d) are shown in yellow.

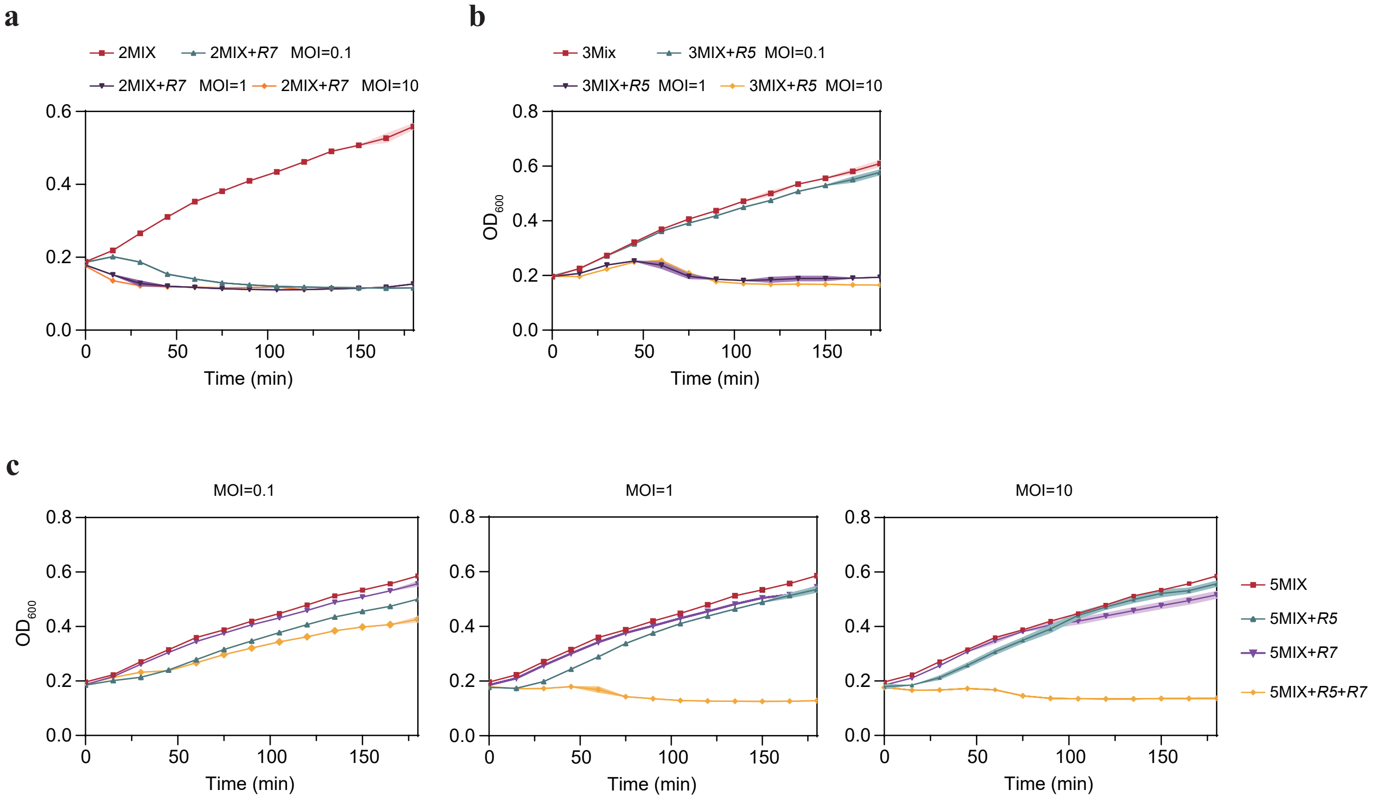

Supplementary Fig. 8. Inhibitory effects of engineered phages used individually or in combination against mixed CRKP cultures representing multiple KL types. (a) Growth inhibition curves of mixed KL25 and KL47 cultures (2MIX) treated with RCIP0109$_{R7}$ at different MOIs. **(b)** Growth inhibition curves of mixed KL2, KL20, and KL112 cultures (3MIX) treated with RCIP0109$_{R5}$ at different MOIs. **(c)** Growth inhibition curves of mixed KL25, KL47, KL2, KL20, and KL112 cultures (5MIX) treated with RCIP0109$_{R5}$ or RCIP0109$_{R7}$ alone or in combination.
